# Screening and Identification of the Host Proteins Interacting with *Toxoplasma gondii* Rhoptry Protein ROP16

**DOI:** 10.3389/fmicb.2017.02408

**Published:** 2017-12-04

**Authors:** Ming Pan, Yanqin Zhou, Yifan Wang, Longjiao Li, Yongle Song, Lun Hou, Junlong Zhao

**Affiliations:** ^1^State Key Laboratory of Agricultural Microbiology, College of Veterinary Medicine, Huazhong Agricultural University, Wuhan, China; ^2^Key Laboratory for Development of Veterinary Diagnostic Products, Ministry of Agriculture, Huazhong Agricultural University, Wuhan, China; ^3^Cooperative Innovation Center for Sustainable Pig Production, Wuhan, China

**Keywords:** *Toxoplasma gondii*, ROP16, protein interaction, Dnaja1, Gabra4, yeast two-hybrid

## Abstract

*Toxoplasma gondii*, as a zoonotic protozoan parasite, develops sophisticated strategies to manipulate hosts for efficient intracellular survival. After successful invasion, *T. gondii* injects many effector proteins into host cells for various purposes. TgROP16 (*T. gondii* rhoptry protein 16), which is secreted from rhoptries into host cells, can activate the host STAT (signal transducer and activator of transcription) signaling pathway through phosphorylation of STAT3 and STAT6. However, whether there are other host proteins modulated by TgROP16 is currently unknown. In this study, yeast two-hybrid (Y2H) screen was used to look for additional host proteins interacting with TgROP16. Yeast cells expressing a mouse cDNA library cloned into the prey vector were used to mate with yeasts expressing ROP16 without signal peptide. Two mouse proteins, Dnaja1 (DnaJ heat shock protein family member A1) and Gabra4 (gamma-aminobutyric acid A receptor, subunit alpha 4) were identified to interact with ROP16 from this screen. Further analysis suggested that the Predomain of ROP16 played key roles in mediating interactions with these host proteins, whereas the contribution from the Kinase domain was minor. The interactions between Dnaja1 and different parts of ROP16 were also estimated *in vivo* by co-immunoprecipitation. The results showed that the Predomain of ROP16 was the major region to interact with Dnaja1, which is consistent with the Y2H results. Based on the gene ontology analysis, Dnaja1 is predicted to participate in stress response while Gabra4 is involved in the system development process. The discovery of new host proteins that interact with ROP16 of *T. gondii* will help us to further investigate the functions of this effector proteins during *T. gondii* infection.

## Introduction

*Toxoplasma gondii*, as a model organism of Apicomplexa parasites, can infect all warm-blooded animals and result in toxoplasmosis worldwide (Montoya and Liesenfeld, 2004; Szabo and Finney, 2017). It’s estimated that one third of the world population has been infected (Tenter et al., 2000; Montoya and Liesenfeld, 2004). As an opportunistic pathogen, it can cause severe disease in immunocompromised patients (Zhou et al., 2011; Zhang N.Z. et al., 2015). Latest studies show that people with HIV infection have a very high burden of *T. gondii* infection worldwide (Wang et al., 2017). As such, *T. gondii* is a big threat to the health of humans and animals (Jiang et al., 2015).

Over the last two decades, significant progress has been made in understanding the pathogenesis of *T. gondii*. Upon invasion, *T. gondii* parasites modify host cells intensively to make them a cozy home for their proliferation using various types of secretory proteins (Koshy et al., 2012). GRA and ROP proteins released from dense granules and rhoptries are the major parasite proteins to manipulate host gene transcription and protein expression. These include ROP5, ROP16, ROP18, and GRA15 etc (Hunter and Sibley, 2012; Cheeseman and Weitzman, 2015). ROPs play vital roles in mediating parasite-host interactions. Studied have found that ROP5, along with ROP17 and ROP18, controls virulence by blocking IFN-γ activated autonomous immune clearance mechanisms in infected host cells (Behnke et al., 2012; Etheridge et al., 2014). ROP5, ROP18, and ROP17 form complex structures on the parasitophorous vacuole membrane (PVM) to phosphorylate IRG and prevent their accumulation on the PVM (Niedelman et al., 2012). Rhoptry protein ROP16, on the other hand, can directly localize to host cell nucleus and activate the host STAT signaling through phosphorylation of STAT3 and STAT6 (Yamamoto et al., 2009; Ong et al., 2010; Butcher et al., 2011; Hunter and Sibley, 2012), leading to prolonged activation of these two transcription factors and subsequent upregulation of interleukin-4 (IL-4) to antagonize IL-12 induction (Butcher et al., 2011; Hunter and Sibley, 2012). Further study have shown that ROP16 of type I and type III strains could sustain the STAT activation response but not the type II strain (Saeij et al., 2007). Moreover, ROP16 immunization could trigger a strong humoral and cellular responses against *T. gondii* (Yuan et al., 2011) and promote host resistance to oral infection and intestinal inflammation in mice, in the context of endogenous GRA15 expression (Jensen et al., 2013). These studies suggest that *T. gondii* ROP16 is an important virulence factor participating in modulation of host gene transcription and protein expression. However, besides STAT3 and STAT6, the host proteins modulated by TgROP16 are currently unknown.

Yeast hybrid systems are widely used for the detection of protein–protein and protein–small molecule interactions. Among various yeast hybrid systems, Y2H is the most commonly practiced, to detect protein–protein interactions and to screen complex genetic libraries for the identification of interacting partners for bait proteins (Legrain and Rain, 2014). It can also be modified to study the interactions of multiple proteins by reformative system (Pause et al., 1999). Many studies have used this tool to discover host proteins that interact with *T. gondii* secretory proteins, which include TgMIC2, TgROP18, TgGRA15 etc (Cheng et al., 2012; Wang et al., 2014; Liu et al., 2017).

To further identify host proteins interacting with TgROP16, we use Y2H method to screen a mouse brain cDNA library. Through this approach, we identified two mouse proteins, Dnaja1 and Gabra4, to interact with the Predomain of TgROP16, which forms new basis to elucidate the functions and mechanisms of ROP16 more comprehensively.

## Materials and Methods

### Parasite and Cell Lines

*Toxoplasma gondii* type I strain RH was propagated in Human Foreskin Fibroblast (HFF) cells. Tachyzoites were harvested and purified by filtration through 3 μm membranes (Whatman Inc., United States) before total RNA extraction. Seven-week-old female Kunming mice, were purchased from the Center for Disease Control (CDC) of Hubei Province in China for mice brain tissue RNA extraction. HEK293T cells were cultured in RPMI 1640 medium (Hyclone, United States) supplemented with 10% FBS (Gibco, United States) and 1% penicillin/streptomycin (Gibco, United States).

### Plasmid Construction

Total RNA was extracted from purified RH tachyzoites using TRIzol Reagent (Invitrogen, United States) according to manufacturer’s instructions. Then one microgram of total RNA was reverse transcribed to cDNA using the ReverTra Ace reverse transcription kit (TOYOBO, Japan). The bait plasmids expressing different parts of TgROP16 were constructed by cloning one of the following three fragments of ROP16 into the pGBKT7 vector: the 2055 bp fragment encoding the full length mature ROP16 (with signal peptide removed, 684 amino acids, from amino acid R24 to M707), the 1056 bp fragment encoding the Predomain (352 amino acids, from amino acid R24 to A375) and the 999 bp fragment encoding the Kinase domain (332 amino acids, from amino acid L376 to M707). Using the cDNA above as templates, these three fragments were PCR amplified using specific primers m*ROP16*-F (5′-GAATTCCGATACATGTCGTTTGAGGAAGC) and m*ROP16*-R (5 ′-GTCGACGCTACATCCGATGTGAAGAAAG) for full length mature ROP16; *ROP16-Pre*-F (5′-GAATTCCGATACATGTCGTTTGAG) and *ROP16-Pre*-R (5′-GTCGACAGCGATCGGCACT) for the Predomain; *ROP16-Kinase*-F (5′-GAATTCCTATACAATCGTGGGCAC) and *ROP16-Kinase*-R (5′-GTCGACCTACATCCGATGTGAAGA) for the Kinase domain. These PCR products were then digested with EcoRI and SalI, and then ligated into similarly digested pGBKT7.

For co-immunoprecipitation (Co-IP) assays, the three fragments of ROP16 mentioned above were tagged with Myc epitope and cloned into the pcDNA3.1 vector. The primers used to amplify these fragments for cloning were listed as follow: pCDNA3.1-*ROP16*-F (5′–GGAATTCATGGAGGAGCAGAAGCTGATCTCAGAGGAGGACCTGCGATACATGTCGTTTGAGGA) and pCDNA3.1-*ROP16*-R (5′-CCGCTCGAGCTACATCCGATGTGAAGAAAGTT) for Myc tagged full length mature ROP16; pCDNA3.1-*ROP16*-F and pCDNA3.1-*ROP16-Pre*-R (5′-CCGCTCGAGCTAAGCGATCGGCACTCCGTTGG) for Myc tagged Predomain of ROP16; pCDNA3.1-*ROP16-Kinase*-F (5′-GGAATTCATGGAGGAGCAGAAGCTGATCTCAGAGGAGGACCTGCTATACAATCGTGGGCACCT) and pCDNA3.1-*ROP16*-R for Myc tagged Kinase domain of ROP16. The corresponding PCR products were digested with EcoRI and XhoI sites, and cloned into pCDNA3.1 vector.

To clone the *Dnaja1* gene from mouse, total RNA was extracted from mouse brain and reverse-transcribed into cDNA as above. Subsequently, the Dnaja1 gene was amplified from cDNA using *Dnaja1*-F (5′-GCTAGCGCTACCGGACTC AGATGGGCAAGGACTACTATCA) and *Dnaja1*-R (5′-TCCTCGCCCTTGCT CACCATTATGGGAAGAACCTGCTCCA) as primers, and was cloned into pEGFP-N1 using ClonExpress homologous recombinase (Vazyme Biotech, Co. Ltd, China). The linearized vector for such cloning was generated by amplification of pEGFP-N1 using primers pEGFP-N1-F (ATGGTGAGCAAGGGCGAGGA) and pEGFP-N1-R (CTGAGTCCGGTAGCGCTAGC).

### Autoactivation and Toxicity Test

To test autoactivation and toxicity of bait protein TgROP16 in yeasts, bait plasmids containing the above mentioned three fragments of ROP16 were individually transformed into *Saccharomyces cerevisiae* strain Y2H Gold according to the Yeastmaker Yeast Transformation System 2 User Manual (Clontech Laboratories, Inc., Mountain View, CA, United States). Transformants were then plated onto agar plates containing appropriate selection media, including SD/-Trp, SD/-Trp/X (40 μg/ml X-α-Gal) or SD/-Trp/X/A (40 μg/ml X-α-Gal and 125 ng/ml Aureobasidin A). The plates were incubated at 30°C for 3 days and the growth (color and size) of transformants were recorded. The empty vector pGBKT7 was included as a control.

### Western Blotting

To check the expression of ROP16 in yeasts, Y2H Gold cells transformed with pGBKT7-mROP16 (a single clone) were inoculated into SD/-Trp broth and cultured until OD_600_ ≈ 0.6. Then total proteins were extracted using the Urea/SDS method (Printen and Sprague, 1994; Wang et al., 2014) and subsequently separated on 12% SDS-PAGE gels and transferred onto PVDF membrane (Millipore, United States). Expression of ROP16 was probed with a mouse anti-Myc antibody (Medical and Biological Laboratories Co. Ltd., Japan), followed by an HRP-conjugated goat anti-mouse secondary antibody (Beyotime Institude of Biotechnology, China). The blot was developed using the direct ECL chemiluminescent method (GE Healthcare, United States).

### Y2H Screen

*Saccharomyces cerevisiae* Y2H Gold and Y187 strains, as well as the medium were purchased from Clontech Co. (Mountain View, United States) for Y2H screen. The Mate & Plate Universal Mouse (Normalized) cDNA library (Clontech, Mountain View, United States) was cloned into the pGADT7-RecAB vector and expressed in Y187 yeast cells. To screen host proteins interacting with ROP16, Y2H Gold cells expressing ROP16 and Y187 cell expressing mouse proteins were mixed to mate for 20–24 h at 30°C. Subsequently the mated cultures were plated onto SD/-Leu/-Trp plates supplemented with 40 μg/ml X-α-Gal and 125 ng/ml Aureobasidin A (DDO/X/A) and incubated at 30°C for 3 days, part of the mated cultures were also plated on SD/-Trp, SD/-Leu, SD/-Trp/-Leu (DDO) plates to calculate the number of clones and the mating efficiency according to manufacturer’s instructions. Afterwards, blue colonies were picked up and tested on higher stringency SD/-Ade/-His/-Leu/-Trp/X-α-Gal/AbA (QDO/X/A) plates.

To identify the genes in positive hits, prey plasmids in positive hits were extracted using TIANprep yeast plasmid DNA kit (TIANGEN Co. LTD., Beijing, China), then sequenced by T7 primer and 3′ AD primer. Sequencing results were blasted with NCBI databases to obtain gene information. Meanwhile, according to GO Classifications, molecular function and biological process of the prey genes were performed by searching the Mouse Genome Informatics (MGI) database^1^ to further estimate the function of the mouse proteins.

### Verification of Protein Interactions by Y2H

To further confirm the potential positive hits, bait plasmids containing ROP16 and prey plasmids were transformed into Y2H Gold and Y187 strains, respectively, according to manufacturer’s instructions for small-scale transformation. Transformants were cultured on SD/-Trp and SD/-Leu plates for 3 days at 30°C and single clones picked from each plate were used for mating. Mated heterozygotes were spread on SD/-Leu/-Trp/X-α-Gal (DDO/X) and QDO/X/A plates and grown for 3–5 days at 30°C. The empty bait vector pGBKT7 without ROP16 was used as a negative control.

To study the interaction between the prey protein and different domains of TgROP16, different fragments of ROP16 cloned in pGBKT7 and prey plasmids were transformed into Y2H Gold and Y187 strain, respectively. Singles clones were mated and tested as above.

### Co-immunoprecipitation

To verify the identified protein–protein interactions *in vivo*, plasmids pcDNA3.1 containing different parts of ROP16 and prey plasmid pEGFP-N1 containing mouse gene Dnaja1 were co-transfected into HEK293T cell for Co-IP assay. Bait and prey plasmids were mixed at the ratio of 1:1 and transfected into HEK293T cells using Lipofectamine 2000 (Invitrogen, United States) according to the manufacturer’s instructions. Empty vectors were included as controls. The tranformants were grown for 28–36 h, harvested and lysed by ultrasonication in RIPA buffer (Beyotime Institude of Biotechnology, China) containing 1 mM phenylmethylsulfonyl fluoride (Sigma–Aldrich Co. LLC., United States) and protease inhibitor cocktail (Sigma–Aldrich Co. LLC., United States). Supernatants of cell lysates were incubated with mouse anti-Myc tag antibody (Medical and Biological Laboratories Co. Ltd., Japan) overnight, followed by Protein G agarose beads (Beyotime Institude of Biotechnology, China) incubation at 4°C for 6 h. Subsequently, the agarose beads were washed with RIPA buffer and proteins bound to agarose beads were solubilized in 1× SDS-sample buffer containing dithiothreitol and boiled for 10 min. Using Western blotting method, ROP16 was detected by mouse anti-Myc tag antibody and the bound Dnaja1 were detected by mouse anti-GFP (Proteintech Group Inc., United States). GAPDH detected by a mouse monoclonal antibody (Proteintech Group Inc., United States) was included as a loading control.

## Results

### Autoactivation, Toxicity, and Expression of ROP16 Baits in Y2H Gold Strain

**FIGURE 1:**
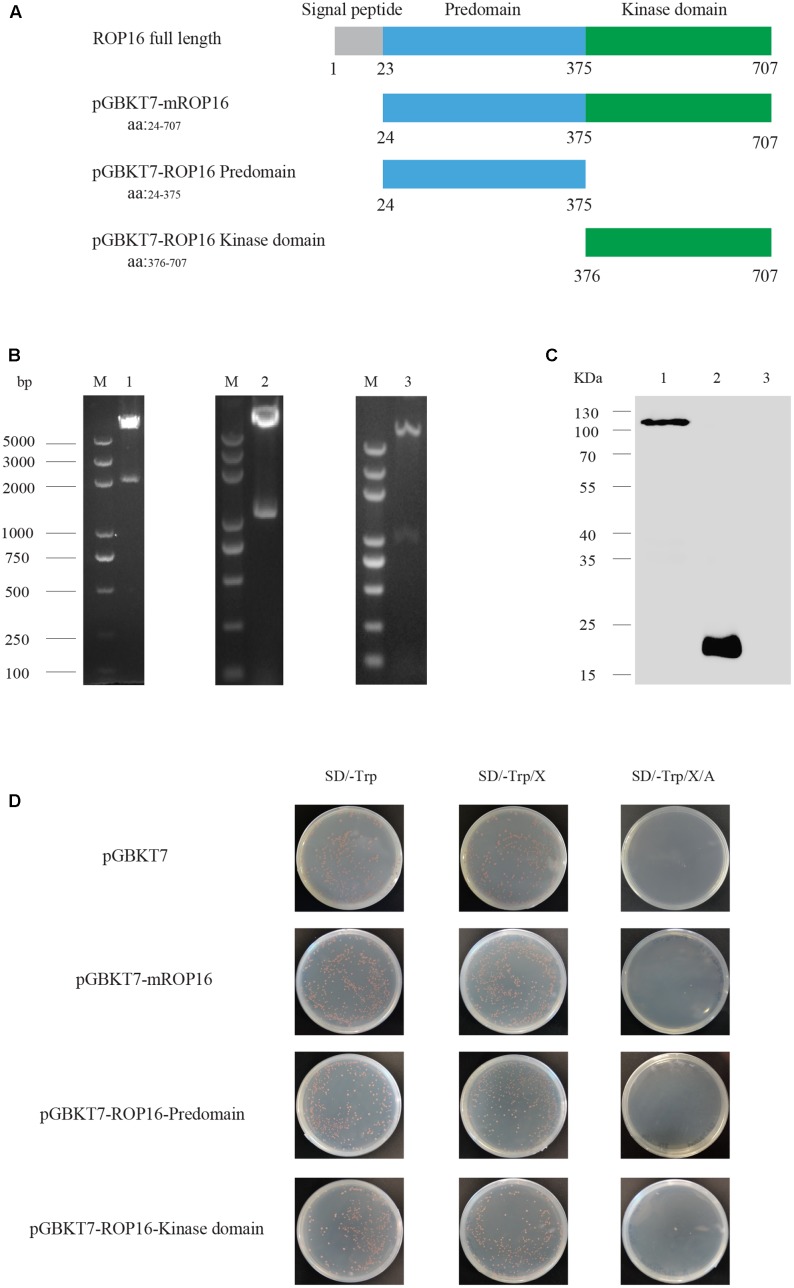
Autoactivation, toxicity and expression tests for ROP16 expressing bait plasmids in yeast cells. **(A)** Schematic illustration of ROP16 full length and different domains of ROP16, mROP16, ROP16-Predomain, and ROP16-Kinase domain. **(B)** Agarose gel electrophoresis of bait plasmids digested with EcoRI and SalI. M: DNA marker 2K plus; Lane 1: pGBKT7-mROP16; Lane 2: pGBKT7-ROP16-Predomain; Lane 3: pGBKT7-ROP16-Kinase domain. **(C)** Western blotting analysis of ROP16 bait expression in yeast cell detected by mouse anti-Myc antibodies. Lane 1: Y2H Gold/pGBKT7-mROP16; Lane 2: Y2H Gold/pGBKT7; lane 3: Y2H Gold cell. **(D)** Detection of autoactivation and toxicity of bait plasmids with SD/-Trp, SD/-Trp/X, and SD/-Trp/X/A indication plates.

To screen for mouse proteins interacting with *T. gondii* ROP16, three different fragments of ROP16, including the mature full length ROP16 without signal peptide (mROP16), the Predomain (ROP16-Predomain) and the Kinase domain (ROP16-Kinase) (**Figures 1A,B**) were amplified from cDNA of RH tachyzoites and cloned into the pGBKT7 vector. These ROP16 expressing constructs were individually introduced into yeast cells and used as baits for two-hybrid screens. Before the screen, the autoactivation and toxicity of ROP16 in yeasts were estimated. To do that, bait plasmids expressing mROP16, ROP16-Predomain, ROP16-Kinase domain were transformed into Y2H Gold yeast cells, respectively. Transformants were grown on SD/-Trp, SD/-Trp/X, or SD/-Trp/X/A plates (**Figure 1D**). Toxicity was indicated by poor growth on SD/-Trp plates, whereas autoactivation was determined by growth of colonies on SD/-Trp/X/A plates and blue colonies on SD/-Trp/X plates. Compared with the empty vector pGBKT7 control, all bait plasmids transformed yeast cells were growing well on SD/-Trp plates. Colonies on SD/-Trp/X plates were white or orange colored instead of blue. No colony growth on SD/-Trp/X/A plates was observed (**Figure 1D**). Taken together, these data indicated that no obvious autoactivation and toxicity were detected for ROP16 baits.

To further check the expression of ROP16 bait in yeasts, Y2H Gold cells transformed with mROP16 or the empty vector were subject to Western blot analysis. Total proteins were extracted from transformed cells by the Urea/SDS method. Western blotting analysis was performed using a Myc-tag antibody. The results indicated that mROP16 and control protein DNA-BD were expressed successfully in Y2H Gold cells (**Figure 1C**). The molecular weight of the mROP16 bait protein was approximately 111 kDa composed of mROP16 protein (90 kDa) fused to the DNA-BD protein (21 kDa), which was consistent with what was detected on the Western blot.

### Y2H Screen

**FIGURE 2:**
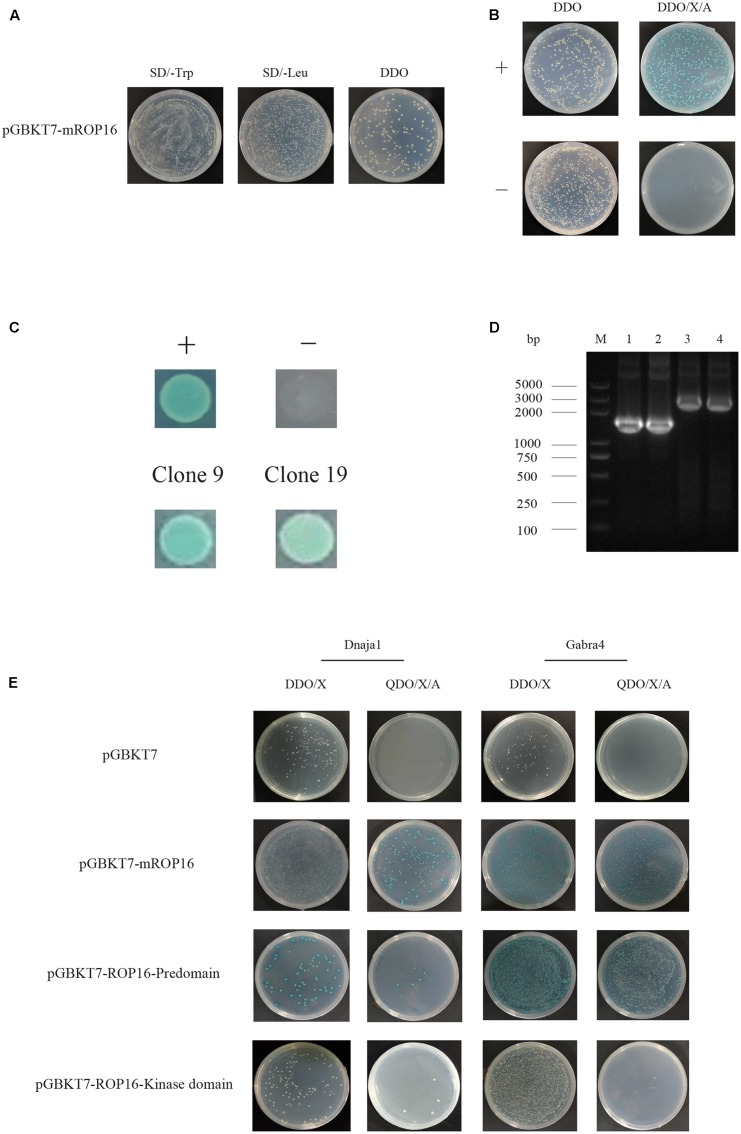
Screen of mouse proteins interacting with mROP16 using yeast two-hybrid (Y2H). **(A)** Determination of mating efficiency. Equal amount of mated cultures (Y2H Gold/mROP16 + Y187/mouse cDNA library) were plated on SD/-Trp, SD/-Leu, and SD/-Trp/-Leu (DDO) plates. Mating efficiency was calculated by dividing the number of colonies on DDO plate by the number of colonies on SD/-Trp or SD/-Leu plates, whichever is less (In this example, there are less colonies on SD/-Leu plate than on SD/-Trp plate, therefore the number of colonies on SD/-Leu plate was used for mating efficiency calculation). **(B)** Growth of positive (+, pGBKT7-53 and pGADT7-T) and negative (-, pGBKT7-Lam and pGADT7-T) controls on Y2H DDO and DDO/X/A indication plates. **(C)** Growth of two positive clones (#9 and #19) on QDO/X/A plates, + and – are positive and negative control colonies, respectively. **(D)** PCR amplification of the inserts in positive clones. M: DNA Marker. Lane 1–2: Clone 9; Lane 3–4: Clone 19. **(E)** Interaction between the two hits and different domains of ROP16. Dnaja1 or Gabra4 expressing plasmids and different domains of ROP16 were transformed into Y187 and Y2H Gold cells, respectively. Transformants were used to mate and grown on DDO/X and QDO/X/A plates to monitor protein interactions.

To screen the host proteins that interact with TgROP16, Y187 yeast cells expressing a mouse cDNA library were used to mate with Y2H Gold cells expressing mROP16, as described in Section “Materials and Methods”. According to the manufacturer’s instruction, mating efficiency was calculated to be 13.3%, while 4.6 × 10^7^ viable diploid yeast cells were screened with proper controls (**Figures 2A,B**). From the colonies grown on DDO/X/A plates, thirty blue clones were selected and tested on higher stringency QDO/X/A plates. Two clones (Clones #9 and #19) out of thirty still maintained blue (**Figure 2C**) while the other 28 failed to give blue colonies, indicating that two potential positive hits were obtained.

### Identification of Mouse Genes in Positive Hits

Plasmids in the two positive clones were extracted and PCR amplified using the T7 primer and 3′ AD primer to estimate the sizes of inserts. Gels electrophoresis results indicated that clone #9 contained a 1.5 Kb insert, whereas clone #19 had an insert of 2.1 Kb (**Figure 2D**). Further sequencing of the inserts and Blast analysis showed that clone #9 contained the *Dnaja1* (AB028273) gene and clone #19 contained the *Gabra4* (NM_010251) gene. According to GO classification, Dnaja1 likely participates in biological processes (cell death, establishment of localization, protein metabolic process, and response to stimulus) and performs molecular function like carbohydrate derivative binding, enzyme regulation, and receptor binding. Gabra4, on the other hand, may be involved in system development as a receptor or a transporter.

### Verification of Identified Hits by Y2H

To further verify these two clones, prey plasmids extracted from them were individually transformed into Y187 cells. Subsequently transformants were used to mate with Y2H Gold cells expressing mROP16 and plated on DDO/X and QDO/X/A indication plates. Growth of blue colonies on these plates implied corresponding protein interactions. As shown in **Figure 2E**, both Dnaja1 and Gabra4 did interact with full length ROP16 as blue colonies were observed on both DDO/X and QDO/X/A plates.

To examine the interactions between Dnaja1/Gabra4 and each domain of ROP16, ROP16-Predomain and ROP16-Kinase domain expressing plasmids were transformed into Y2H Gold cells, respectively, and mated with Dnaja1 or Gabra4 expressing Y187 cells. Mated cultures were plated on the above-mentioned indication plates. The results showed that Dnaja1 mainly interacted with full length ROP16, as blue colonies were only observed during mROP16 mating (**Figure 2E**). Dnaja1 also interacted with the Predomain of ROP16. But the interaction was probably not as strong as with full length ROP16, since less blue colonies were observed during ROP16-Predomain mating (**Figure 2E**). Gabra4, on the other hand, interacted with both full length and the Predomain of ROP16 (**Figure 2E**).

### Validation of Protein Interactions by Co-IP

**FIGURE 3:**
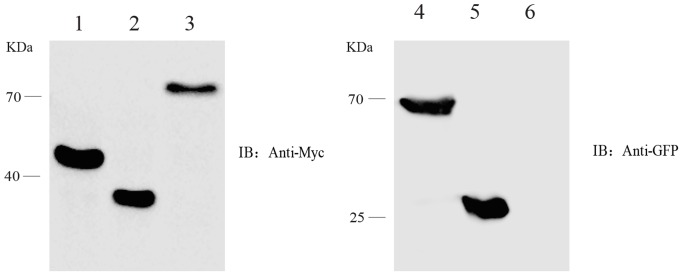
Expression of different parts of ROP16, as well as Dnaja1 in HEK293T cells. HEK293T cells transfected with ROP16 expressing plasmids or Dnaja1 expressing plasmids were subject to Western blotting analysis 24 h post transfection. ROP16 fragments expressed from pCDNA3.1 were detected by anti-Myc tag antibody and Dnaja1 expressed from pEGFP-N1 was detected by an anti-GFP monoclonal antibody. Lane 1: pCDNA3.1-ROP16-Predomain; Lane 2: pCDNA3.1-ROP16-Kinase domain; Lane 3: pCDNA3.1-mROP16, Lane 4: pEGFP-N1-Dnaja1; Lane 5: pEGFP-N1; Lane 6: empty HEK293T cell.

**FIGURE 4:**
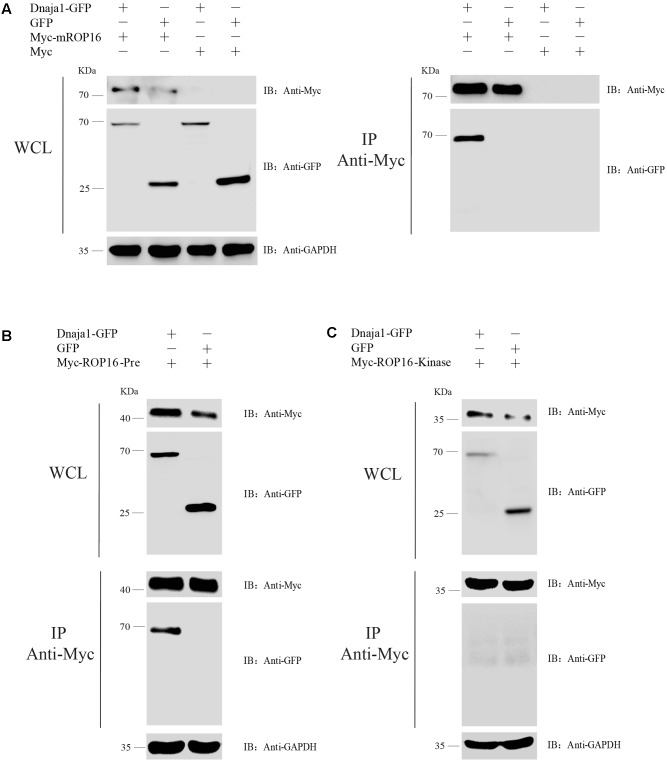
Verification of the interaction between ROP16 and Dnaja1 by co-immunoprecipitation. **(A)** Co-transfection of Myc-mROP16 and Dnaja1-GFP expressing plasmids, as well as control plasmids in different combination, into HEK293T cells and whole cell lysates (WCL) were probed with anti-Myc, anti-GFP, and anti-GAPDH (internal control). Subsequently the lysates were precipitated with an anti-Myc antibody and probed with an anti-GFP antibody to check the precipitation of Dnaja1-GFP. **(B,C)** Interaction between Dnaja1 and Predomain **(B)** or Kinase domain **(C)** of ROP16 determined by Co-IP, similar to **(A)**.

In order to verify the interactions between ROP16 and host proteins *in vivo*, Dnaja1 was chose to be co-expressed with different domains of ROP16 in HEK293T cells and assess their interactions by Co-IP. First, expression of each of these proteins in HEK293T cells were examined. To do this, pCDNA3.1 containing different fragments of ROP16 was individually transfected into pCDNA3.1 and then transfected into HEK293T cells using Lipofectamine 2000, and expression of ROP16 fragments was determined by Western blotting using an anti-Myc antibody 24 h post transfection. Results in **Figure 3** showed that all three fragments of ROP16 could be well expressed in HEK293T cells. Similarly, Dnaja1 was also expressed well in HEK293T cells as a GFR fusion (pEGFP-Dnaja1) and detected by anti-GFP monoclonal antibodies (**Figure 3**). To assess protein interactions by Co-IP, each of the ROP16 fragment expressing plasmids was mixed with pEGFP- Dnaja1 at the ratio of 1:1, and co-transfected into HEK293T cells. Cell lysates prepared from transfectants 24 h post transfection were immunoprecipitated with an anti-Myc antibody. The GFP-Dnaja1 protein bound to precipitated ROP16 was detected by an anti-GFP antibody. Consistent with the Y2H results, GFP-Dnaja1 was co-precipitated with full length mature ROP16, as well as the Predomain of ROP16 (**Figures 4A,B**). Dnaja1 did not show obvious co-precipitation with the Kinase domain of ROP16, consistent with the lack of interaction of the two (**Figure 4C**). The interactions detected by Co-IP were likely to be specific, since negative controls using GFP alone or Myc tag alone did not give detectable Co-IP products (**Figure 4**). Together these results indicated that Dnaja1 was able to be interact with full length, as well as Predomain of ROP16.

## Discussion

Previous studies have reported that *T. gondii* rhoptry protein ROP16 was involved in the activation of host STAT signaling pathway when it reached host cell nuclei (Hunter and Sibley, 2012). STAT3 and STAT6 were shown to be phosphorylated by ROP16 (Yamamoto et al., 2009; Ong et al., 2010; Butcher et al., 2011), but different alleles of ROP16 exhibited different capacities in activating STAT signaling (Saeij et al., 2007). Of the three clonal strain types (type I/II/III), all strains were able to induce STAT3 and STAT6 activation initially, but only type I and type III strains sustained this response and type II strains did not. In this study, a high throughput Y2H screen was used to search for host proteins interacting with ROP16 of type I strain RH. Two proteins, Dnaja1 (AB028273) and Gabra4 (NM_010251) were identified from a mouse cDNA library to interact with ROP16. The interactions between Dnaja1 and different parts of ROP16 were further estimated by Co-IP and the results showed that the Predomain of ROP16 was the major domain to mediate such interactions. Co-IP was also tried to assess the interaction between Gabra4 and ROP16, however, no informative results were obtained because the expression of Gabra4 in HEK293T cells was below the detection limit. The reason for this low expression is currently unknown. Due to the limitations of the yeast two hybrid approach, our screen is probably not yet exhausted because known TgROP16 interactors such as STAT3 and STAT6 were not identified. In addition to the common limitations of yeast two hybrid screen, another important factor that determines the outcome of our screen is the host cDNA library. The one used in our screen is derived from mouse brain, which may have different gene expression patterns than other tissues. This may partially explain why only two proteins were identified from our screen. In this regard, further work is needed to screen libraries derived from other tissues to estimate the overall interactions between ROP16 and host proteins.

Dnaja1, a member of the HSP40s family proteins, is highly conserved (greater than 95% sequence similarity) in most vertebrates (Orthwein et al., 2012). Many proteins that interacted with Dnaja1 have been identified. In the presence of environmental stress, human Dnaja1 activates a DnaK protein through forming a complex with it, to suppress the JNK pathway and the hyperphosphorylation of c-Jun (Stark et al., 2014). Down-regulation of Dnaja1 in pancreatic cancer cells probably reduced the activity of DnaK even under stress conditions, which promoted the occurrence and progression of cancer due to cell proliferation (Stark et al., 2014). Another study demonstrated that activation-induced deaminase (AID) interacted with Dnaja1 and Dnaja2 *in vitro*, but only Dnaja1 over-expression increased AID levels and biological activities, while depletion of Dnaja1 reduced AID levels in cell lines (Orthwein et al., 2012). Previous studies have revealed that AID contributed to antibody-mediated autoimmune diseases (Zaheen and Martin, 2011) as well as to cancer (Okazaki et al., 2003; Pasqualucci et al., 2008). Thus, Dnaja1 plays a nonredundant role in the stabilization of AID and balancing immunity and cancer development indirectly (Orthwein et al., 2012). Moreover, Dnaja1 was verified as a novel substrate to interact with histone deacetylase 6 (HDAC6) by quantitative proteomic analyses (Zhang L. et al., 2015), while HDAC6 played a role to initiate proper impaired immune response with the model of HDAC6-deficient mouse (Zhang et al., 2008). With the results from this work and previous studies, we speculate that ROP16-Dnaja1 complex may participate in immune response modulation. Further work is needed to dissect the biological significance of this interaction.

*Gabra4*, which encodes the α4 subunit of gamma-aminobutyric acid A receptor, belong to alcohol-regulated genes (ARGs) that show remarkable plasticity in response to alcohol (Suryanarayanan et al., 2011). Previous studies indicated that Gabra was considered to be the primary molecular target of injectable anesthetics (Iyer et al., 2014). It is rapidly up-regulated by acute alcohol exposure and down-regulated in cortical neurons during alcohol withdrawal. Gabra4 was regulated by specific microRNAs (Bekdash and Harrison, 2015), as well as protein kinases (PK) like PKA and PKC (Carlson et al., 2016). Further study have shown that Gabra4 played a critical role in trafficking of the δ subunit to the neuronal surface where both were major mediators of tonic inhibition in the thalamus (Peng et al., 2014). The levels of Gabra4 mRNA and protein were elevated in spontaneous seizures (Grabenstatter et al., 2014). According to the role of Gabra4 in stress response and nervous system activity, ROP16 may affect these processes through interacting with Gabra4.

As a protein kinase, ROP16 is composed of a Predomain and Kinase domian with a signal peptide at the N-terminus (**Figure 1A**). Previous studies demonstrated that type I and type III ROP16 could modify STAT3 by phosphorylation, but type II ROP16 could not. A single polymorphic amino acid (L503S) in the Kinase domain of ROP16 determined STAT3 activation (Yamamoto et al., 2009). On the other hand, little information is available regarding the function of the Predomain. In this study, the interactions between ROP16-Predomain and Dnaja1 was identified through Y2H and Co-IP, indicating important roles of the Predomian of ROP16. Further studies are required to explore the exact functions of ROP16-Predomain during *Toxoplasma* infection.

In summary, two mouse proteins, Dnaja1 and Gabra4 were identified to interact with TgROP16 by Y2H screen. The interaction between ROP16 and Dnaja1 was further verified by Y2H, as well as Co-IP methods. These results provided important clues to further investigate the functions of TgROP16.

## Author Contributions

JZ conceived and designed the study. JZ and YZ revised the manuscript critically. MP performed the studies and wrote the manuscript. YW assisted in the yeast two-hybrid screen. LL, YS, and LH contributed to yeast mating and Co-IP assay. All authors read and approved the final manuscript.

## Conflict of Interest Statement

The authors declare that the research was conducted in the absence of any commercial or financial relationships that could be construed as a potential conflict of interest.

## Footnotes

1 http://www.informatics.jax.org/

## Abbreviations

cDNA: complementary deoxyribonucleic acid
Dnaja1: DnaJ heat shock protein family member A1
Gabra4: gamma-aminobutyric acid A receptor, subunit alpha 4
GO: Gene Ontology
GRAs: Dense granule proteins
HIV: Human immunodeficiency virus
IRG: immunity-related GTPases
NCBI: National Center for Biotechnology Information
PCR: Polymerase chain reaction
ROPs: Rhoptry proteins
STAT: signal transducer and activator of transcription
TgROP16: *T. gondii* rhoptry protein 16
Y2H: Yeast two-hybrid
